# Combining radar and direct observation to estimate pelican collision risk at a proposed wind farm on the Cape west coast, South Africa

**DOI:** 10.1371/journal.pone.0192515

**Published:** 2018-02-06

**Authors:** Andrew R. Jenkins, Tim Reid, Johan du Plessis, Robin Colyn, Grant Benn, Rhonda Millikin

**Affiliations:** 1 *AVISENSE* Consulting, Cape Town, Western Cape, South Africa; 2 Percy FitzPatrick Institute of African Ornithology, DST-NRF Centre of Excellence, University of Cape Town, Cape Town, Western Cape, South Africa; 3 NCC Environmental Services, Cape Town, Western Cape, South Africa; 4 Geocline Consulting, Cape Town, Western Cape, South Africa; 5 EchoTrack Inc., Ottawa, Ontario, Canada; Centro de Investigacion Cientifica y de Educacion Superior de Ensenada Division de Fisica Aplicada, MEXICO

## Abstract

Pre-construction assessments of bird collision risk at proposed wind farms are often confounded by insufficient or poor quality data describing avian flight paths through the development area. These limitations can compromise the practical value of wind farm impact studies. We used radar- and observer-based methods to quantify great white pelican flights in the vicinity of a planned wind farm on the Cape west coast, South Africa, and modelled turbine collision risk under various scenarios. Model outputs were combined with pre-existing demographic data to evaluate the possible influence of the wind farm on the pelican population, and to examine impact mitigation options. We recorded high volumes of great white pelican movement through the wind farm area, coincident with the breeding cycle of the nearby colony and associated with flights to feeding areas located about 50 km away. Pelicans were exposed to collision risk at a mean rate of 2.02 High Risk flights.h^-1^. Risk was confined to daylight hours, highest during the middle of the day and in conditions of strong north-westerly winds, and 82% of High Risk flights were focused on only five of the proposed 35 turbine placements. Predicted mean mortality rates (22 fatalities.yr^-1^, 95% Cl, 16–29, with average bird and blade speeds and 95% avoidance rates) were not sustainable, resulting in a negative population growth rate (λ = 0.991). Models suggested that removal of the five highest risk turbines from the project, or institution of a curtailment regimen that shuts down at least these turbines at peak traffic times, could theoretically reduce impacts to manageable levels. However, in spite of the large quantities of high quality data used in our analyses, our collision risk model remains compromised by untested assumptions about pelican avoidance rates and uncertainties about the existing dynamics of the pelican population, and our findings are probably not reliable enough to ensure sustainable development.

## Introduction

Poorly located commercial-scale wind energy developments can have an adverse effect on local bird populations [1, 2]. While some potential wind farm locations are obviously problematic from the start, in other cases the avian impact issues may not be so immediately clear, and distinguishing good from poor sites may be heavily dependent on the quality of field data collected to inform the environmental authorization process.

The primary objectives of avian impact studies for proposed wind farms are to (i) measure the potential exposure of birds in the receiving environment to possible collision and displacement impacts, (ii) estimate the scale of actual impacts should the wind farm be authorised and built, and (iii) predict the significance of these estimated impacts in terms of the long-term persistence of affected bird populations [1, 2]. The process is inherently subjective and often heavily dependent on largely untested assumptions [3, 4, 5, 6, 7], and yet its outcomes profoundly influence decision-making in the development of utility-scale facilities, and in the spread of an industry currently subject to rapid global growth. For the most part, while pre-emptive impact mitigation based on studies conducted before construction is clearly critical to ensuring truly sustainable development, most effective mitigation is delivered by detailed post-construction work, designed to reduce harmful impacts of wind farms that are already operational [8, 9, 7].

South Africa has one of the highest potentials for wind energy development in Africa [10]. In keeping with similar documents guiding such work in other parts of the world, the South African best practice guidelines for assessing wind farm impacts on birds [11] generally advocate a simple, low-cost, observer-based approach to quantifying bird movements through development areas, but acknowledge that accumulating enough data in this way to do meaningful collision risk assessments is difficult, and that the spatial accuracy of such data is generally low [12,13]. An alternative is to use remote sensing devices such as radar to gather much larger quantities of more accurate spatial data, although identifying and separating out the tracks of particular groups or species of birds from all the radar’s tracked targets, which may be critical to the outcomes of an avian impact study, can be challenging [14]. Even if this is possible, and a relatively accurate estimate of possible collision risk for a given priority species is achieved, the value of this measure is still questionable in the absence of good, local demographic data for that species, needed to gauge the actual population-level effects of predicted wind farm-related mortality rates [15, 16].

**Fig 1:**
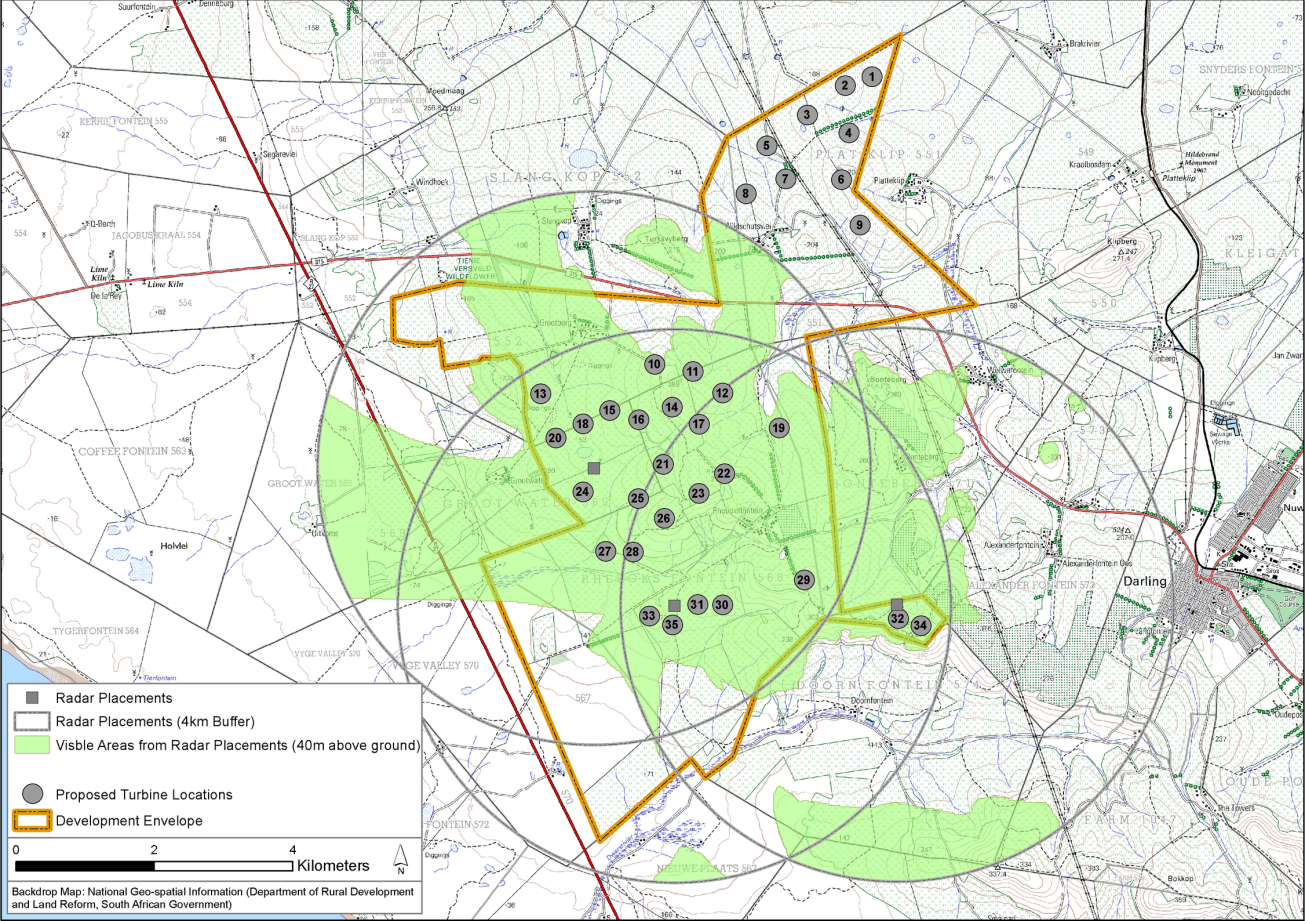
Proposed wind farm location and radar coverage achieved for tracking pelicans. General situation of the study wind farm, and the theoretical and effective viewsheds of the radar from the three placements used to track great white pelican movements through the development area, in relation to the proposed turbine layout. Basemap reprinted from 1:50 000 topographic maps 3318AC Yzerfontein, 3318AD Darling and 3318 CB Melkbosstrand, under CC BY license, with permission from the South African Government Printers (Authorisation No. 11778, dated 25 August 2017), original copyright 1978.

We studied the possible effects on birds of a wind farm development proposed for a site on the south-western coast of the Western Cape Province, South Africa (Fig 1). The site is situated close to Dassen Island, which hosts the only breeding colony in the region of the great white pelican *Pelecanus onocrotalus* [17]. By integrating observer- and radar-based flight path data with relatively high quality demographic information, we delivered a uniquely detailed assessment of the possible impacts of a planned wind farm on this population of threatened birds.

## Materials and methods

The proposed wind farm development area is located between the rural town of Darling and the coastal town of Yzerfontein, about 15 km east of Dassen Island (which lies 8 km off the coast at Yzerfontein) (Fig 1). If built, the completed facility will be contained within an area of about 10 x 8 km, and will comprise 35 x 3 MW wind turbines, each of which will have a hub height of 120 m and a rotor diameter of up to 126 m. The area features open, hilly heathland (altitude averages about 160 m above sea level, rising to just over 300 m at the highest point), and the local climate is temperate, featuring warm, dry, windy summers, and wetter, cooler winters.

Direct observations completed at the development site in 2012–2013 indicated that commuting great white pelicans frequently flew directly through the proposed turbine-populated area, and potentially within the height range of the rotor sweep. A risk model based on this initial sample of directly observed pelican passage rates through the site suggested that turbine collision mortality would be unsustainable. However, although this work was done in full compliance with the best practice guidelines for assessing wind farm impacts [11], the findings were compromised by small sample sizes and wide confidence intervals attending estimated pelican collision rates [4].

In order to better understand avian collision risk issues at the site, and to explore mitigation options that might reduce the impact of the proposed wind farm (e.g. layout adjustments, operational management including turbine curtailment options), we studied the pelican: wind farm interface in greater detail. Survey effort was focused on great white pelican movements only, and the sampling regime included the deployment of radar to accumulate more data with sufficient three-dimensional accuracy to more reliably estimate the predicted collision risk for pelicans, and to explore possible ways to minimize this risk.

### Fieldwork

Data were collected by a team of at least two fieldworkers, in six sampling periods spread over eight months in 2013/14 (Sampling period: (1) Late winter, 24 July– 01 August; (2) Early spring, 03–13 September; (3) Late spring, 22–27 October; (4) Early summer, 26 November-01 December; (5) Late summer, 28 January– 02 February; (6) Early autumn, 27 February– 04 March). Each replicate included two full days and one full night of radar deployment at each of three tracking locations. This sampling regimen allowed for good baseline coverage of bird movements through the development area in relation to the full spread of the pelican breeding season in late July to early March [18], and a representative range of weather conditions.

Each site visit included time allocated to an observer-based approach, involving (i) the opportunistic accumulation of pelican movement data from a range of vantage points scattered around the general area, and (ii) the compilation of a baseline of co-observations to calibrate and refine the radar tracking data. Vantage point watches involved both observers searching for and recording details of pelican movements through the area; whenever possible, flight paths were plotted on 1:50 000 hard-copy maps, and were later digitized and converted into a GIS dataset.

Co-observations were made from each radar placement, and involved one of the fieldworkers visually scanning the airspace around the radar for signs of approaching pelicans, while the second watched the radar screen. A minimum of 3 h of co-observation was done daily, spread randomly through the day in periods of at least 1 h. Data recorded included details of the corresponding radar track, to facilitate coupling of the two sets of information. Given that the pelicans are so large and easily detectable, and moved through the site relatively slowly and infrequently, this simple, manual method of validating the radar information was considered adequate for the purposes of this study [19, 20].

We also conducted counts of pelicans arriving at and leaving the Dassen Island colony over two days at the height of the breeding season (06–07 November 2013), synchronized with radar coverage at the study site, and did opportunistic counts (*n* = 5; 04 and 05 September, 07 November, 05 December 2013, and 26 February 2014) of pelicans feeding at and roosting nearby the Vissershok Waste Management Facility, located about 60 km to the south-east, which was the primary destination of foraging pelicans travelling south through the study area. On-site fieldwork was conducted on private land, with the express permission of the landowner. Additional fieldwork on Dassen Island and at the Vissershok Waste Management Facility was done with the permission of CapeNature and the management of the waste management facility respectively. No hands-on or destructive sampling was done during this study.

### Radar deployment

We used the EchoTrack^TM^ omni-directional radar-acoustic sampling system to record the flight patterns of all airborne-wildlife in the area [21]. The radar used was a 25 kW, 3 cm wavelength surveillance Raccal Decca BridgeMaster E, with a 1.8 m X-band antenna, modified to provide coverage of target height in addition to range and azimuth [22], a range resolution of 7.5 m, an angle resolution of 0.5°, and a height accuracy of 15 m [16]. The radar capture volume included a 3–4 km radius around each placement (Fig 1), with a maximum altitude of 1950 m. Each of the three radar placements (T1-3) was selected to maximize coverage of the southern two-thirds of the proposed development area (Fig 1), where earlier work had shown that pelican activity was greatest.

Using the EchoTrack software, the flight path or track of each flying target was defined by a set of consecutive track points (geographic coordinates with corresponding heights above ground, recorded every 1–3 s). The radar discriminated individual birds except where flocks of pelicans were flying at a distance to the radar, or very close together. Flocks were divided into two categories: 2–10 birds or >10 birds.

To protect against loss of data, recording was paused for 1 min every 15 min for data storage. As a result, long flights through the study area were broken into segments. Radar data were correlated with site-specific weather (temperature (°C), wind speed and direction) throughout each sampling period. using a Kestrel™ 4500 weather station, positioned on a tripod 1.5 m above the ground about 30 m from the radar.

### Data processing

All flight path information was collated in a central database. Radar tracks were matched with corresponding visual observations based on congruence in distance from the radar, time of day, and direction of flight. Each radar track was given the attributes of flight mode and flock size from the co-observed pelican flight. The remaining, remotely obtained radar data were identified as pelicans using classification trees [23–25] derived from the parameters defining co-observed radar tracks. The model-derived identification criteria were then applied to all radar tracks (with or without visual co-observations) to infer the total sample of great white pelican flight tracks through, over and around the development area.

These filtered data were uploaded to a graphical information system (GIS) using ArcMap 10.2 (ESRI, Redlands, California). Track points were considered to be located within the rotor sweep of any given turbine placement if they fell within a spherical volume centred on the turbine hub and extended to the radial length of the blade (63 m), buffered by 17 m to account for the three-dimensional accuracy of the radar.

Pelican tracks were termed “High Risk” flights if they comprised points located within the buffered rotor sweep (BRS) of any planned turbine/s. Where radar tracks were allocated to one of the two coarse flock size categories, it was generally assumed that these groups of birds functioned as a unit (with the movements of the rear birds dependent on those of the lead), and each flock was treated as one observation. However, for calculations of collision rate, each individual was seen as a potential casualty and was treated as a separate track. In order to convert single tracks by flocks to multiple individual flights, each such record was multiplied by the mean flock size (for its respective category) recorded by direct observation during the relevant sampling period.

#### Modelling pelican flights through the wind farm

Generalised Linear Models (GLMs) were then used to explore this database, using a two-stage approach. First, the accumulated radar coverage was broken into 10 min observation segments, with each segment characterised in terms of various temporal and conditional variables, and the aggregate number of pelicans tracked through the study area and through the BRS over that period. This dataset was used to model the volume of pelican traffic observed in the general area of the wind farm. The count data attributed to each 10 min observation period comprised positive, whole numbers, consistent with a Poisson process, but about two-thirds of the observations included no pelicans at all, so the full dataset was over-dispersed. Ultimately, the data were found to fit a negative-binomial distribution (which can be treated approximately as a Poisson with an over-dispersion factor instead of a constant probability) and were modelled accordingly, yielding results on a log scale rather than on the scale of the original observations. A refined suite of environmental factors was built into the model, including time of year (sampling period), time of day, wind direction, wind speed and air temperature. These variables were considered to be those which were (i) most likely to influence pelican numbers in the area, and (ii) most usefully inform a mitigation strategy for the wind farm. Time of day and wind direction were modelled as categories to simplify interpretation. Two random terms were added–date (because it was assumed that pelican behaviour would be more similar within days than between them), and a means to identify each 10 min observation period. The latter acted as an over-dispersion factor in R [26] and was required to model the data as a negative binomial.

Second, the likelihood that pelicans flying in the vicinity of the wind farm would actually pass through the BRS was estimated in terms of the full complement of recorded pelican tracks. With the number of pelican flights as the number of observations, and the number of High Risk flights as the number of outcomes, the tracking data approximated a binomial distribution. However, a true binomial distribution treats the probability of there being an outcome as uniform for all observations. Examination of the pelican data suggested that this probability varied between observation periods and iterations, so it was estimated using a beta-binomial distribution [27], in which the probability of an outcome is modelled with two parameters so that it can vary. These parameters were estimated using the mean and the variance of observed probabilities. In using a beta-binomial distribution, the mean estimates remain the same, while the estimated confidence intervals are wider (i.e. the estimated range of predicted values is greater). It was assumed that High Risk flights were influenced by similar variables to those affecting overall pelican traffic volumes, but with the addition of the currently proposed turbine layout for the wind farm project (with each turbine placement a function of the effects of underlying topography—ground height, slope and slope aspect). The results of the two models were combined to derive estimates of the numbers of High Risk flights likely to occur per annum in relation to the current proposed wind farm layout, and then under a variety of spatial, temporal and conditional mitigation scenarios.

#### Collision risk assessment

The mean numbers of High Risk flights by great white pelicans through the aggregate BRS of the wind farm were converted to predicted mortality rates in terms of various possible collision and collision avoidance scenarios [3]. The Band model [3], was used to calculate collision rates in terms of pelican interactions with the southern two-thirds of the proposed development only (27 of 35 turbines), during daylight hours only (average = 12 h.day^-1^), and over the period mid-July to mid-March only (about 243 days or 2920 daylight hours), since previous observations, supported by the findings of this study, indicate that great white pelicans do not commute through the northern third of the project, do not regularly fly at night, and do not aggregate at and travel to and from Dassen Island over the non-breeding period in mid-March to mid-July [18].

The probability of a bird flying through the BRS being hit by the blades was calculated using various parameters (S1 Table) and calculations set out in the specified spreadsheet recommended by the Band model [3], with the models run under low, average and high bird and rotor speed scenarios. For each model, the probability of collision was averaged from the two provided under gliding and flapping flight modes (flight mode alters the model calculations slightly), as pelicans were observed to do both.

Given that birds are likely to take action to avoid collisions, either by being completely displaced from the area, or by flying above, below or around turbines on approach, the model outputs must be adjusted accordingly. Observed avoidance rates of other large bird species generally vary from 95% to 99% [3, 8, 28], so we estimated impacts for both 95% and 98% avoidance rates. However, given the large size and weight of the great white pelican—wing-span up to 3.6 m, mass up to 15 kg [29]—it may be highly collision prone [30], and the more conservative figure of 95% may be more appropriate.

#### Population-level impacts

The potential effects of collision mortality on the great white pelican population were examined using a simple, age-structured Leslie matrix population model [31, 32], built using the available demographic data (S2 Table; and assuming that the starting condition was a population close to a stable age distribution), and a parameter for collision mortality. As is usual in such models, it was assumed that females limit the population’s growth and only this sex was modelled. Sensitivity to maximum age was tested by comparing a truncated model (maximum age of 30 –S2 Table) and a non-truncated model (old birds can remain in the oldest age class). There were few birds in older age classes so varying this parameter had little influence on the population growth rate (λ). To be conservative with respect to the impacts of collision mortality, non-truncated models were used which required a higher mortality rate to materially affect the population. In the absence of collision data, it was assumed that turbine mortality would be equal across ages and sex.

Great white pelican breeding performance in this area is known to be highly variable, in terms of both the proportion of the population breeding in a given year (S1 Fig), and the number of chicks successfully raised [18]. In light of these ongoing fluctuations, the demographic impacts of the proposed wind farm were modelled for three breeding scenarios (low, medium and high).

## Results

### Observed behaviour and movements

The field team accumulated 180.6 h of direct observation time, including co-observation (65.2 h) and vantage point (115.4 h) observations, and recorded 407 great white pelican flocks commuting through the area, totalling 4539 birds (S3 Table). In 185 (45.5%) of these instances, the pelicans were using environmental sources of lift (soaring or thermaling) to gain altitude when sighted and for most of the time they were observed, whereas in 156 (38.3%) they were passively gliding cross-country, and in only 66 (16.2%) they were using powered, flapping flight to cover ground.

**Fig 2:**
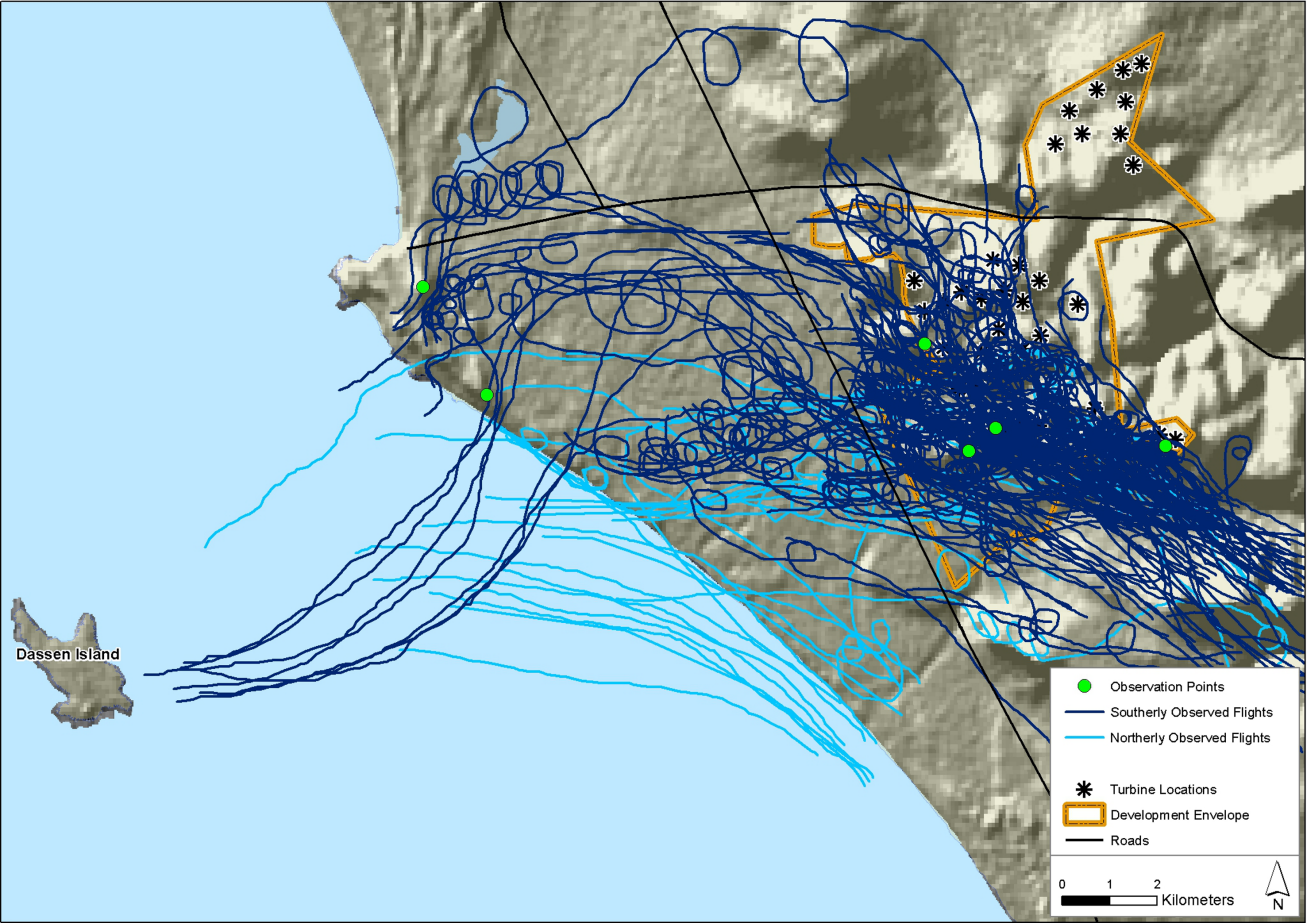
Observed pelican flight paths, and the locations of vantage points and proposed turbines. Approximate paths of pelican flights observed in the study area during periods of vantage point and co-observation work, in relation to the locations of observation points from which the data were collected, and the proposed layout of the study wind farm. These flight paths were digitized from plots made by eye on hard-copy 1:50 000 topographical maps. Basemap reprinted from 1:50 000 topographic maps 3318AC Yzerfontein, 3318AD Darling and 3318 CB Melkbosstrand, under CC BY license, with permission from the South African Government Printers (Authorisation No. 11778, dated 25 August 2017), original copyright 1978.

The observed flight paths (*n* = 158) typically either headed south along the coastal plain from Dassen Island, or north along the coastal plain when returning to the island (Fig 2). Most flights (79%, *n* = 158), and the vast majority of south-bound flights (90%, *n* = 127) passed directly through the proposed wind farm development area, while most north-bound flights (65%, *n* = 31) remained close to the coastline, away from the turbine layout. Very few flights were observed heading north from the wind farm site, and no pelicans were seen to encroach on the northern-most array of nine turbines (Fig 2).

In addition to the observation time accumulated in the proposed development area, 15.3 h were spent observing pelicans as they arrived at and left the breeding colony on Dassen Island. Two counts of these movements, conducted on consecutive days, recorded 265 commuting flocks of pelicans, totalling 1706 birds. In the more complete count, conducted from 06h30 to 16h00 on 07 November, 621 birds arrived at the island from the mainland in 76 flocks (mean flock size = 8.2 birds, range = 1–150), and 590 birds departed the island in 101 flocks (mean flock size = 5.8 birds, range = 1–61). The timing of these movements (both inbound and outbound) was concentrated in the middle of the day, between 10h00 and 14h00 (S2 Fig).

Incidental counts of great white pelicans in the vicinity of the Vissershok Waste Management Facility showed that significant numbers of birds were feeding and roosting in this area throughout much of the study period, with numbers varying from >300 birds in early September to >1100 birds in early December.

### Radar-tracked movements

A total of 588.7 h of radar coverage was logged at the three placements over the study period (including 415.1 daylight hours–S4 Table), during which nearly 1.8 million track points were recorded, describing >85 000 wildlife flight paths. Radar tracks were matched with corresponding great white pelican co-observations with a level of accuracy (99%, 95%, or 80% respectively) based on distance, time and direction disparities from the closest co-observed record. The model used to filter pelican flight tracks from other airborne wildlife was built from 729 matched cases (*n*_*1*_ = 489 co-observed training observations, *n*_*2*_ = 240 test observations), and confirmed visual identifications for 92.1% of great white pelicans. The model-derived identification criteria were then applied to all radar-derived flights (with or without visual co-observations) and the final sample of pelican tracks (*n* = 14 999 tracks from all radar deployment time, comprising 415 545 track points, *n* = 14 459 tracks from the six full sampling periods, comprising 402 095 track points) was assigned an independent level of accuracy of 97%.

**Fig 3:**
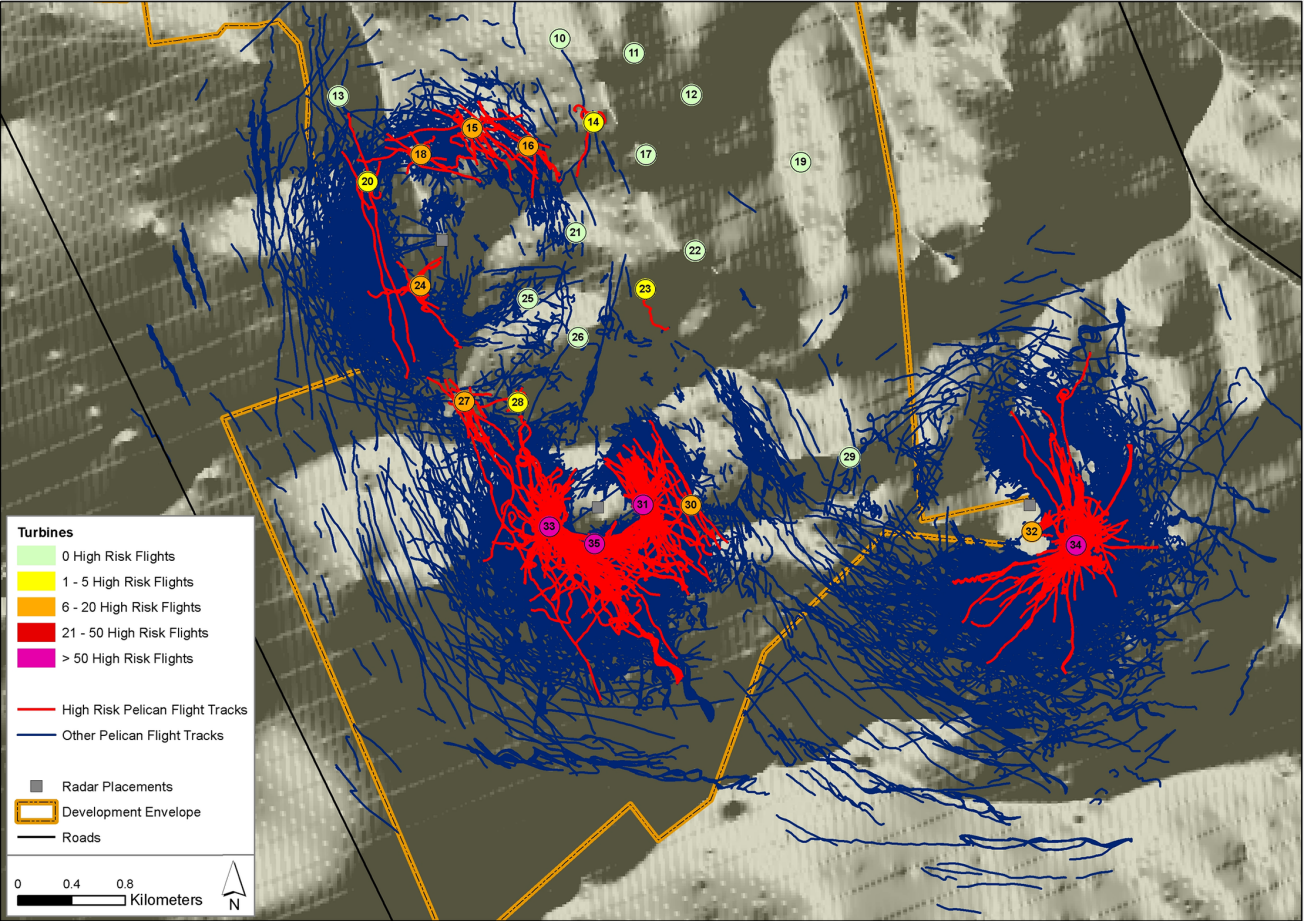
Pelican flight tracks recorded by the radar over the full study period. All great white pelican flight tracks recorded by the radar over the full study period, plotted on a map of the current project layout, with High Risk flights (those intersecting with the BRS) shown in red, and the turbine placements colour-coded according to predicted collision risk. Basemap reprinted from 1:50 000 topographic maps 3318AC Yzerfontein, 3318AD Darling and 3318 CB Melkbosstrand, under CC BY license, with permission from the South African Government Printers (Authorisation No. 11778, dated 25 August 2017), original copyright 1978.

Of the tracks obtained from the six full sampling periods, 589 passed through the BRS and were classed as High Risk (Fig 3). In 1085 (7.5%) of these, the radar data suggested that >1 bird was involved (in 608 instances flock = 2–10 birds, and in 477 instances flock > 10 birds). By multiplying each of these tracks by the mean observed flock size in each category per sampling period (S3 Table), the total sample of individual pelican tracks used to derive estimates of collision rate was 28 783, of which 710 were classed as High Risk. Note that in the subsequent analyses, track points that were > 1000 m (~5%) were ignored, since these instances compromised the normality of the dataset and were located well outside of the potential impact area.

### The incidence of High Risk flights

#### Volumes of pelican traffic

The data accumulated over the six full sampling periods were summarized into 4062, 10 min observation periods (S4 Table). The GLM outputs show that pelicans were present in the study area in greatest numbers during sampling period 2 (Spring), between 09h00 and 15h00, during conditions of NW wind, and with increasing wind speed and increasing temperature (Table 1).

**Table 1:** GLM outputs of the probability of High Risk flights.

	Degrees of freedom	Deviance	Residual Degrees of freedom	Residual Deviance
NULL			401158	40746
StartPeriod	4	75.49	401154	40671
FlightMode	2	727.74	401152	39943
standHeightAboveGround	1	2311.78	401151	37631
fTrackDirection	3	119.82	401148	37511
fWindDirection	3	100.06	401145	37411
I(WindSpeed—mean(WindSpeed))	1	18.53	401144	37393
I(Temperature—mean(Temperature))	1	0.57	401143	37392
Sampling period	5	161.95	401138	37230
fNearestTurbine	23	2748.06	401115	34482
Sampling period:fNearestTurbine	93	1841.17	401022	32641

Approximate relative importance of variables is shown by the Deviance. Sampling Period, Nearest Turbine, and their Interaction need to be treated together.

#### The probability of High Risk flights

The likelihood of pelicans flying through the BRS was largely a function of the interaction between sampling period (time of year) and the turbine layout. The model outputs (S5 Table) suggest that the probability of collisions taking place at a given turbine placement varied through the year, but that the nature of this variation was not consistent between turbines (S5 and S6 Tables). Overall, High Risk flights occurred at only 15 of the 25 turbines, and were more likely during sampling periods 1 and 3 than in sampling period 2, and less likely during sampling periods 4 and 5. However, these patterns were not consistent (S5 and S6 Tables). The probability of High Risk flights increased with temperature and wind speed, and when the wind was blowing from the north-west, when pelicans were gliding or thermaling from the north-east or south-west, and before 09h00 or after 15h00 (S5 Table).

#### The frequency of High Risk flights

**Fig 4:**
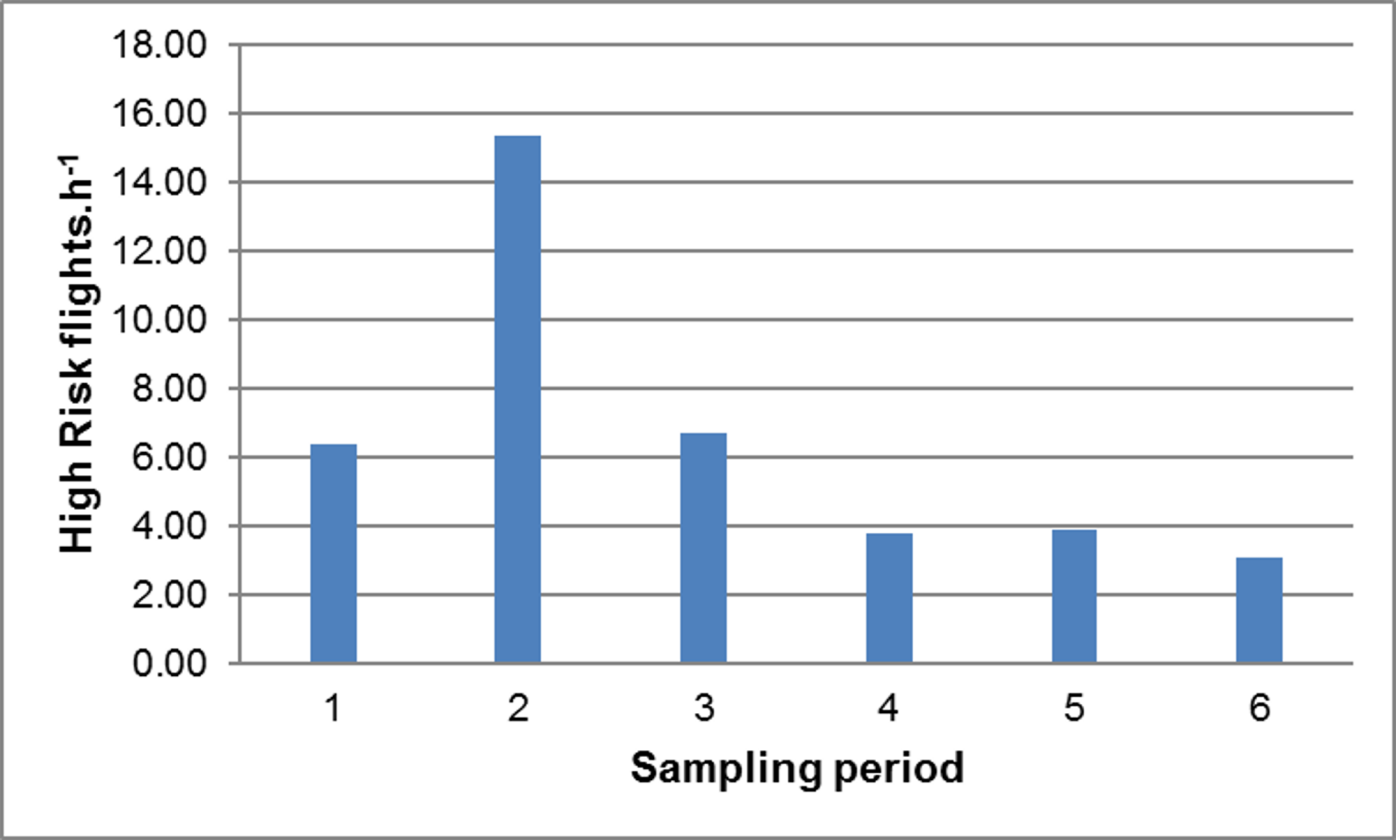
Pelican collision risk *vs* season. Estimated frequency of High Risk flights.h^-1^ in relation to sampling period. In each case, rates were calculated using the GLM with other environmental factors held constant.

**Fig 5:**
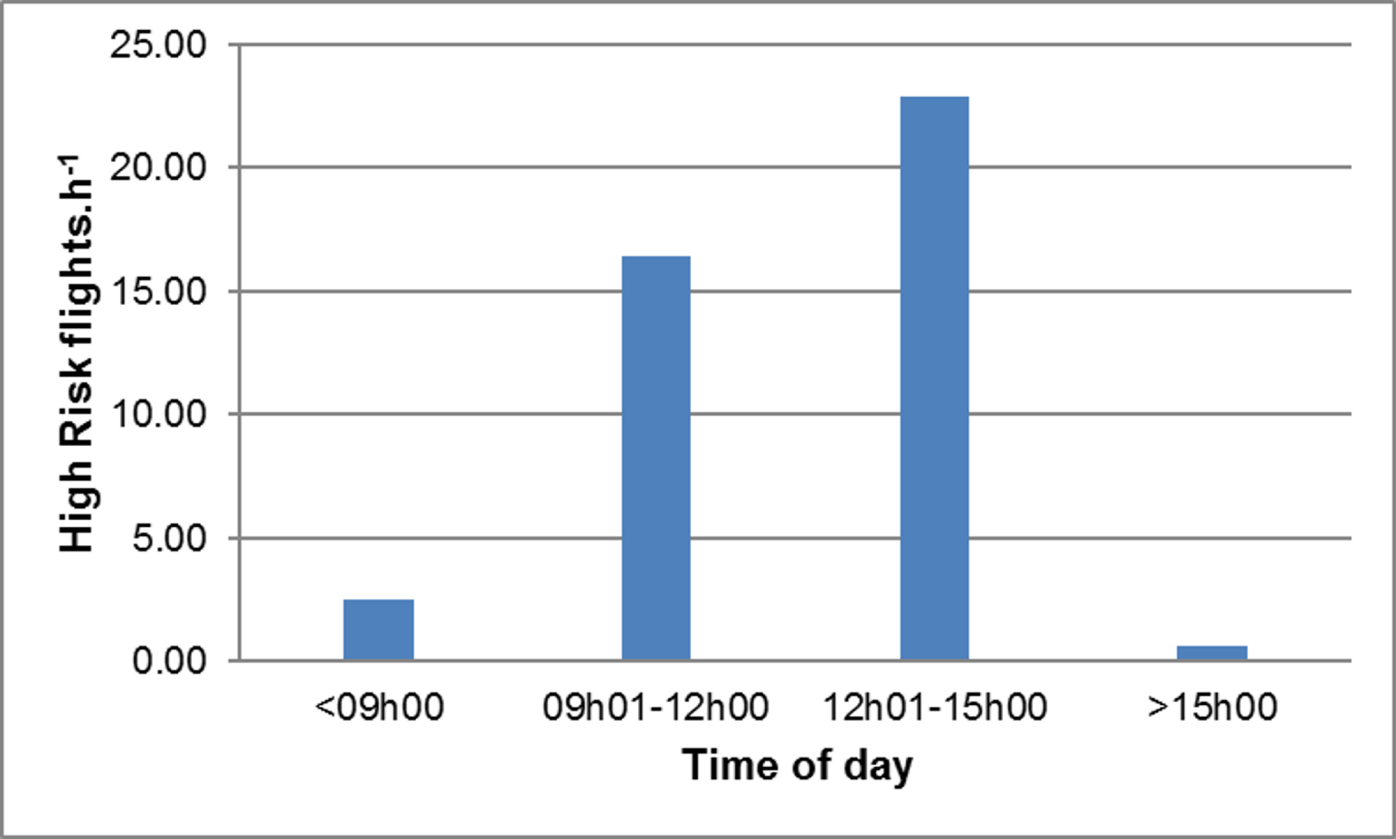
Pelican collision risk *vs* wind direction. Estimated frequency of High Risk flights.h^-1^ in relation to wind direction. In each case, rates were calculated using the GLM with other environmental factors held constant.

**Fig 6:**
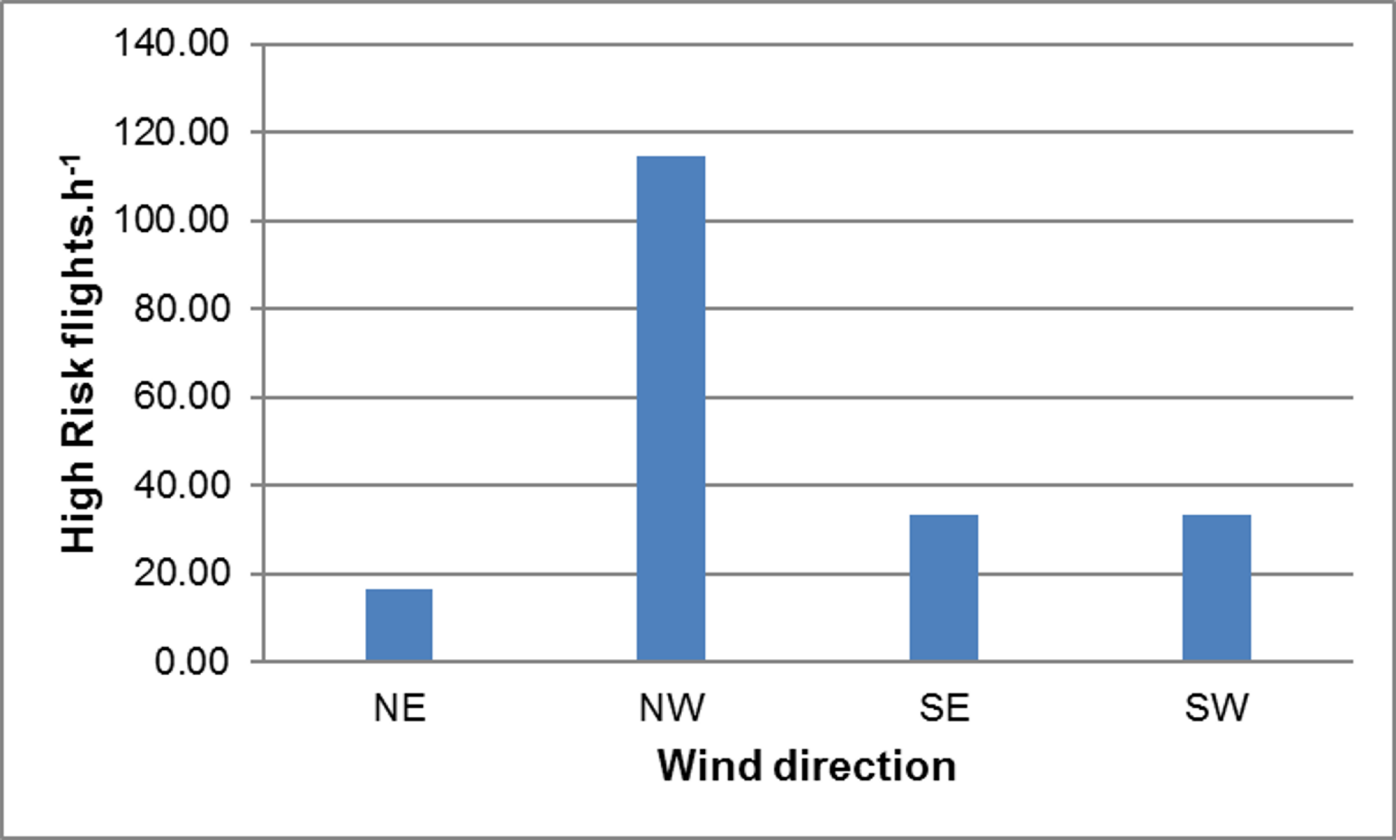
Pelican collision risk *vs* time. Estimated frequency of High Risk flights.h^-1^ in relation to time of day. In each case, rates were calculated using the GLM with other environmental factors held constant.

**Fig 7:**
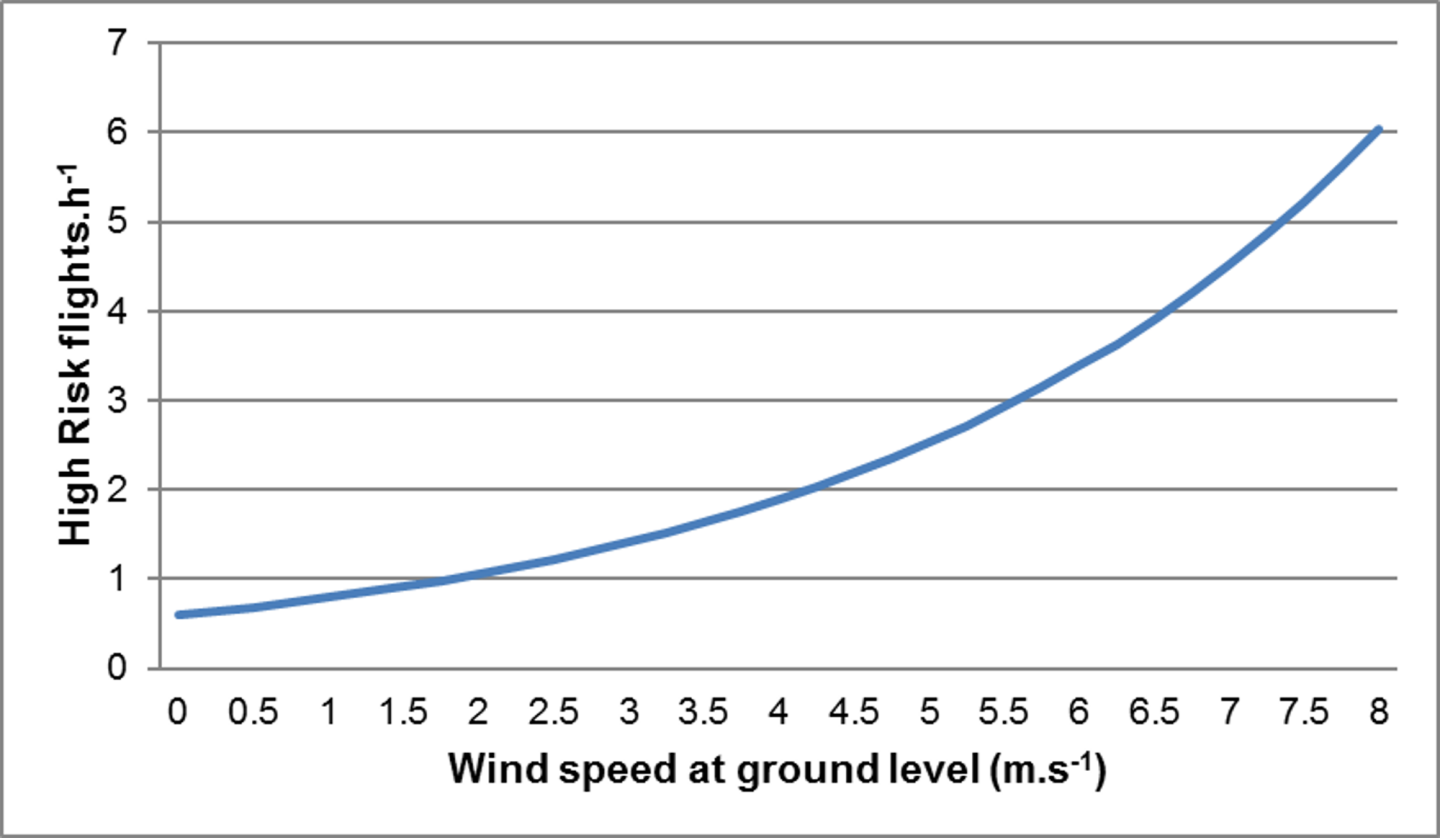
Pelican collision risk *vs* wind speed. Estimated frequency of High Risk flights in relation to wind speed at ground level. Other environmental factors were held constant (Sampling period 2, observation period 09h00-12h00, Wind from the North-East, gliding bird, slope facing east, and mean Ground Height, and temperature).

The frequency of High Risk flights (and hence the predicted frequency of collisions) was calculated as the product of the number of birds present in the vicinity of the wind farm and the probability that these commuting birds would intersect with the BRS. High Risk flights were most frequent during sampling period 2 (Spring) (Fig 4), mainly because of the high volumes of pelican traffic observed at this time (as the probability of any given flight intersecting with the BRS was essentially constant across all sampling periods). Similarly, High Risk flights were most frequent when the wind was from the north-west (mainly because of the high traffic volumes recorded under these conditions) (Fig 5), and between 09h00 and 15h00 (because traffic volumes were highest in the middle of the day) (Fig 6), and with increasing wind speed (both traffic volumes and the proportion of flights through the BRS were higher at higher wind speeds) (Fig 7).

### Predicted collision rates

The mean frequency of High Risk flights over the entire study period was 2.02 flights.h^-1^ (95% Cl 1.46–2.71 flights.h^-1^), which converts to 5898 (4263–7913) High Risk flights annually (over the eight months of the pelican breeding cycle). Allowing for various bird *vs* rotor speed combinations [10], and a range of possible avoidance rates, the predicted pelican collision rate for the proposed wind farm layout ranges from 5–2230, with about 22 great white pelican casualties annually perhaps the most likely outcome (Table 2).

**Table 2:** Modelled annual fatalities of pelicans under various conditions.

Avoidance scenario	Bird speed	Rotor speed		
		Low	Average	High
A. No avoidance	Low	735 (530–985)	1467 (1059–1957)	2230 (1612–2991)
	Average	287 (125–586)	438 (316–585)	609 (441–826)
	High	249 (181–333)	307 (222–413)	394 (283–525)
B. 95% avoidance	Low	37 (27–49)	73 (53–99)	111 (81–149)
	Average	14 (10–19)	22 (16–29)	31 (22–41)
	High	12 (9–17)	15 (11–21)	20 (14–26)
C. 98% avoidance	Low	15 (11–20)	29 (21–39)	45 (32–60)
	Average	6 (4–8)	9 (6–12)	12 (9–16)
	High	5 (4–7)	6 (4–8)	8 (6–11)

Mean (95% CI) numbers of great white pelicans likely to be killed annually in collisions with wind turbines at the proposed Cape west coast wind farm, estimated using the Band model (2007). Results are presented for a range of avoidance levels (A. No avoidance, B. 95% avoidance and C. 98% avoidance), with varying bird and turbine speeds.

### Demographic impacts and mitigation options

**Fig 8:**
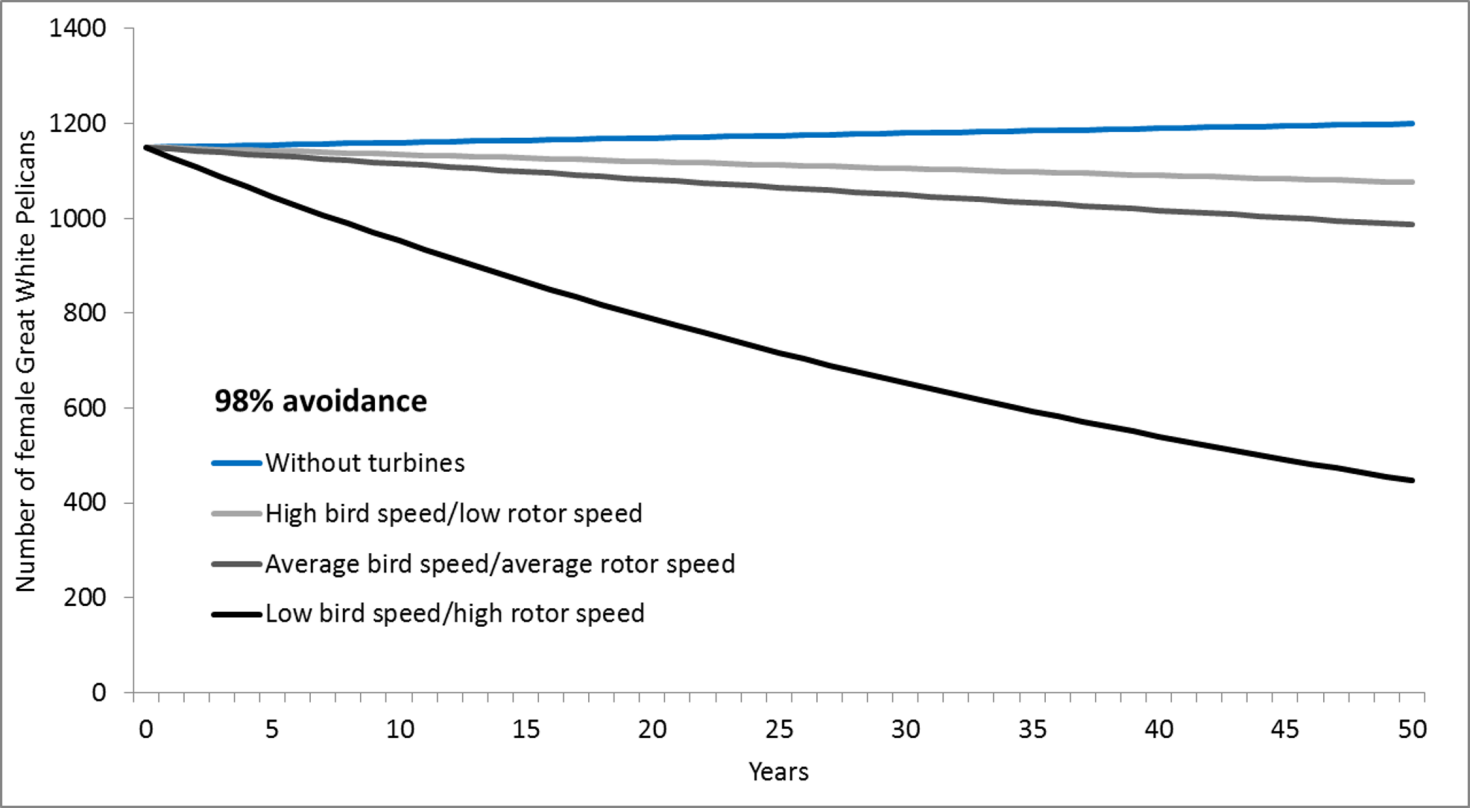
Estimated pelican population trends in response to various turbine collision rates: Higher avoidance. Modelled growth rates of the Dassen Island great white pelican population (with medium breeding success) in response to possible rates of collision mortality, as affected by various bird and rotor speed combinations and a collision avoidance rate of 98%.

**Fig 9:**
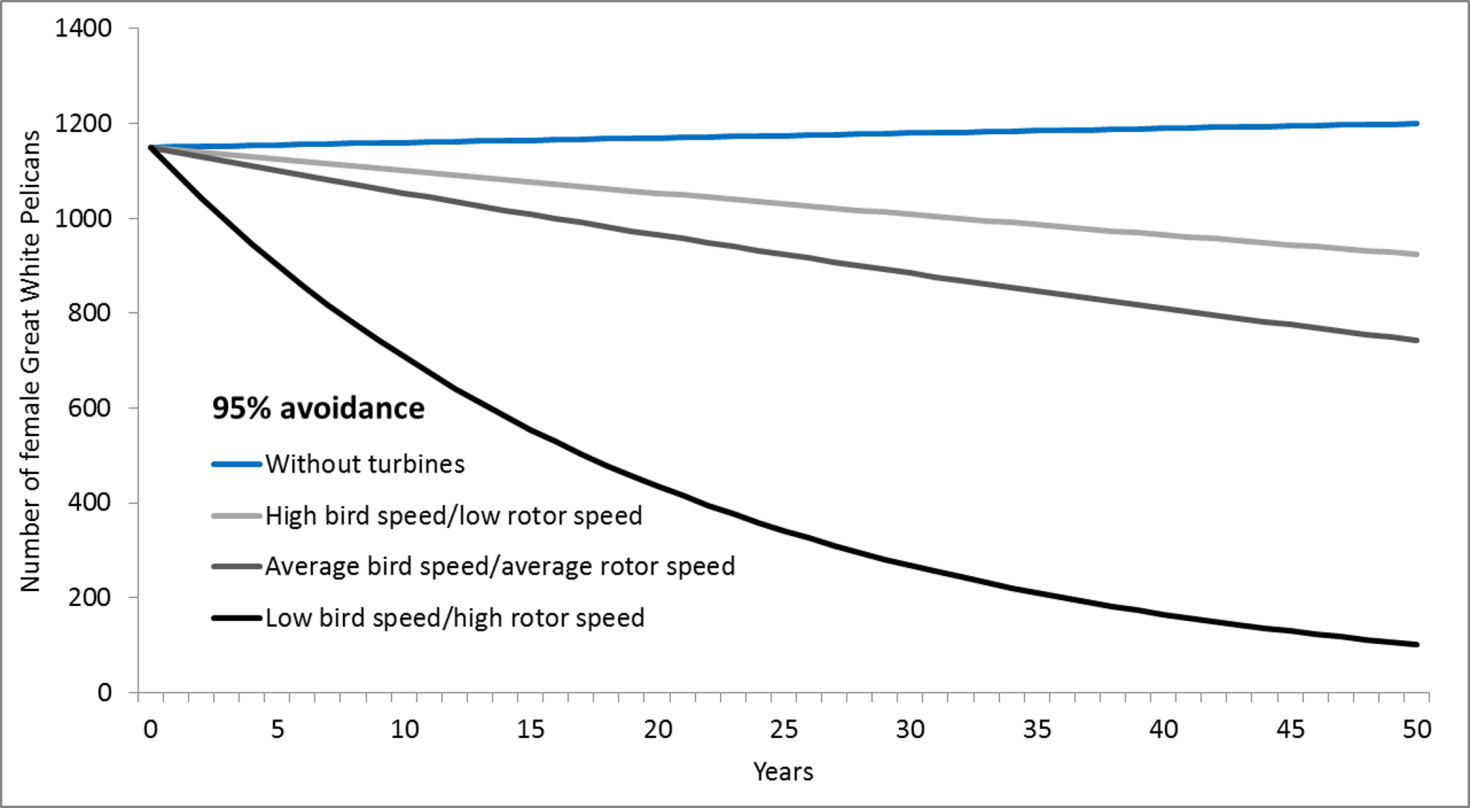
Estimated pelican population trends in response to turbine collision rates: Lower avoidance. Modelled growth rates of the Dassen Island great white pelican population (with medium breeding success) in response to possible rates of collision mortality, as affected by various bird and rotor speed combinations and a collision avoidance rate of 95%.

In the absence of turbines, the Dassen Island great white pelican population is thought to be approximately stable (λ ≥ 1 –Table 3). However, even low levels of collision mortality resulting from the construction of the wind farm could possibly tip the population into decline (λ < 1) (Figs 8 and 9). If breeding success is relatively high, collisions still have the potential to cause a population decrease, and if aggregate collision rates for the population are higher than the tested scenarios (e.g. if combined with other wind energy facilities built in the near vicinity), then the population could go into rapid decline. While there will probably be an associated reduction in pelican traffic and collision rates as the affected population shrinks, the demographic implications of this are probably negligible in relation to the levels of variation already intrinsic to the modelled results.

**Table 3:** Estimated pelican population growth rates under various combinations of bird- and turbine-related parameters.

Collision scenario (% avoidance, bird /rotor speed)	Numbers of female pelicans killed in collisions annually (% mortality)	Breeding scenario (female chicks raised per female in the population annually)		
		Low (0.02)	Medium (0.06)	High (0.12)
*Without turbines*	*0 (0%)*	*0*.*974*	*1*.*001*	*1*.*036*
98%, high/low	2.5 (0.2%)	0.972	0.999	1.034
98%, average/average	4.5 (0.4%)	0.970	0.997	1.032
98%, low/high	22.5 (2.0%)	0.955	0.981	1.017
95%, high/low	6 (0.5%)	0.969	0.996	1.031
95%, average/average	11 (1.0%)	0.965	0.991	1.027
95%, low/high	55.5 (34.8%)	0.926	0.953	0.988

Population growth rate (λ) estimated with Leslie matrix models for great white pelicans under various breeding and wind turbine collision mortality rate scenarios at the proposed wind farm on the Cape west coast. (see S2 Table for demographic data and Table 4 for collision mortality estimates).

**Fig 10:**
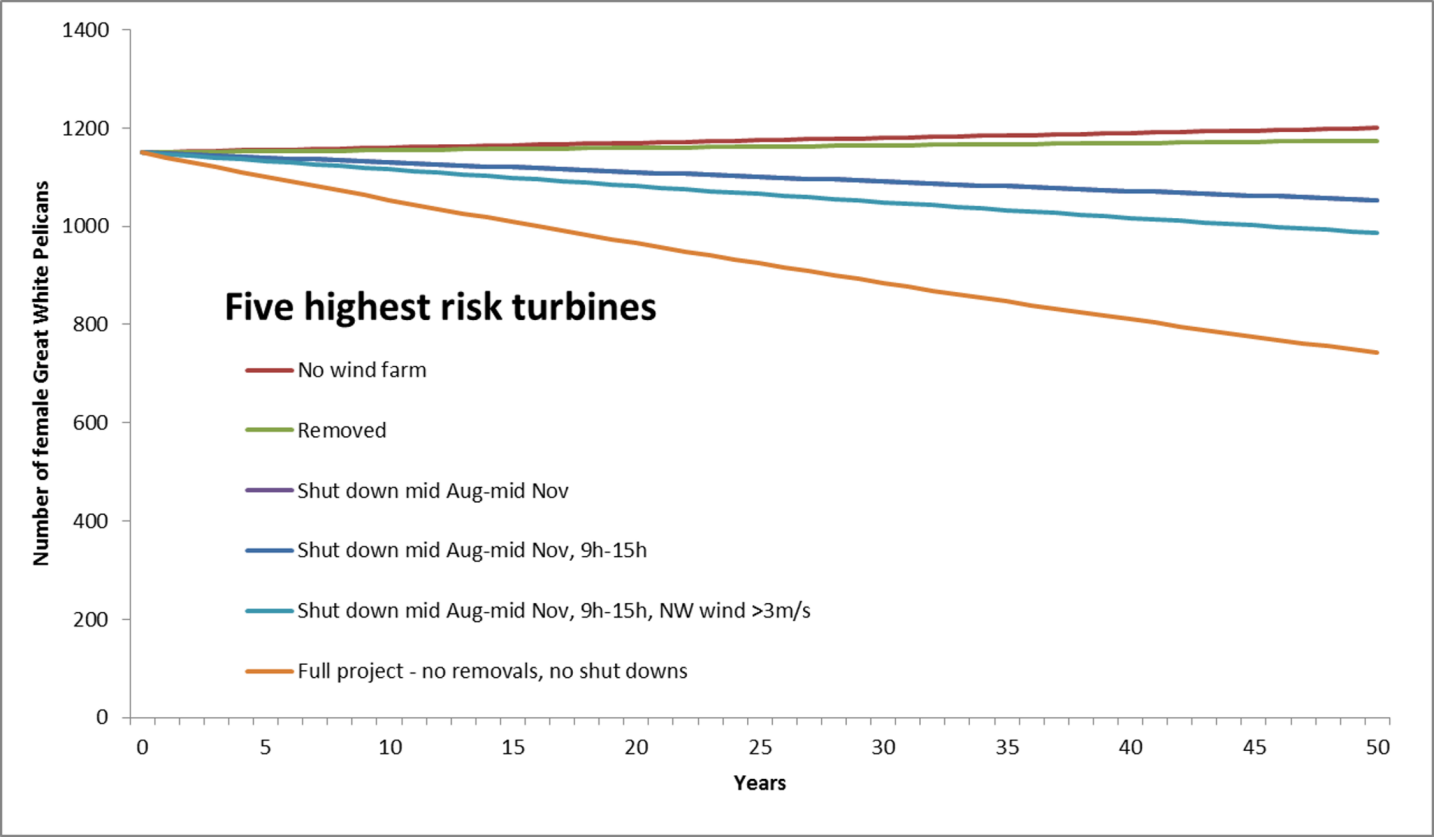
Estimated pelican population trends in response to various turbine collision rates after mitigation. Modelled growth rates of the Dassen Island great white pelican population (with medium breeding success) in response to predicted rates of collision mortality (with 95% avoidance) associated with various mitigation options applied to the five highest risk turbines.

Four possible mitigation options were explored in relation to three combinations of project elements (Table 4). Clearly, the removal of all 15 turbines associated with collision risk (Turbines 34 (166), 33 (164), 31 (105), 27 (78), 35 (60), 14 (25), 18 (19), 15 (17), 32 (13), 20 (13), 24 (12), 16 (11), 30 (8), 28 (7) and 23 (1), listed in decreasing order of the number of pelicans implicated in High Risk flights through the BRS, with the number of flights recorded in parentheses) or the removal of the 10 highest risk turbines (which accounted for 94% of High Risk flights), were the most effective interventions, greatly reducing predicted mortality. Even removal of the five highest risk turbines reduced predicted collision rate by >80%, and seasonal and temporal curtailment of the same suite of machines (for six hours of the day, every day, for three months of the year), lowered collision risk by > 70%, with associated reductions in population-level impacts (Fig 10). In contrast, conditional, temporal and seasonal curtailment (in which allowances were made for wind conditions, in addition to time of year and time of day) was far less effective (Table 4, Fig 10), and probably can be disregarded as a viable approach to mitigation, given the possibility that avoidance rates could be lower than allowed for, and the combination of bird and rotor speed more condusive to greater collision risk.

**Table 4:** Estimated pelican population growth rates after mitigation, under various combinations of bird- and turbine-related parameters.

Targeted project components	Mitigation options			
	Removal	Seasonal curtailment^1^	Temporal curtailment within season^2^	Conditional curtailment at certain times within season^3^
All 15 risk turbines	0	6 (5–7)	6 (5–9)	9 (6–12)
10 highest risk turbines	0 (0–4)	6 (5–7)	6 (5–7)	9 (6–12)
Five highest risk turbines	1 (0–7)	6 (5–7)	6 (5–7)	9 (6–12)

Estimated collision mortality figures (Cl 95%) for great white pelican at the proposed wind farm on the Cape west coast in relation to various mitigation options. Figures are for average bird and rotor speeds and 95% avoidance rates.

^1^ Shut-down mid-August to mid-November.

^2^ Shut-down 09h00-15h00, mid-August to mid-November.

^3^ Shut-down when wind direction NW, wind speed at ground level > 3m.s^-1^, 09h00-15h00, mid-August to mid-November.

## Discussion

### Observer- vs radar-based information

The results of this relatively intensive study largely confirm the findings of the original, more generic collision risk assessment for this proposed wind farm project. However, by quadrupling the coverage achieved of the affected area (in terms of observation time spent at key locations), doubling the amount of pertinent data accumulated (in terms of the number of pelicans observed flying through the development area), hugely increasing the spatial resolution of these data (from rough estimates made by eye to three-dimensional plots located within a 17 m buffer), and subjecting the data to more rigorous statistical interrogation–all improvements made possible by the deployment of radar–these results are far more accurate, and are presented with much greater confidence [4].

The core dataset on which this study is based was collected remotely, and is only as reliable as the methods used and assumptions made in its processing and interpretation [20]. However, it is important to note that broad consistency between these radar-based findings and earlier (as well as coincident) direct observations of pelican flight patterns in the area suggests that the processed radar data are both real and reliable [14]. Also, with the radar positioned to collect critical flight path information at the wind farm site, the team of observers was able to cover ground and record aspects of pelican behaviour beyond the immediate footprint of the development, adding much-needed biological context to the on-site data, and further justifying and validating the remote-sensing approach.

### Patterns of pelican movement

**Fig 11:**
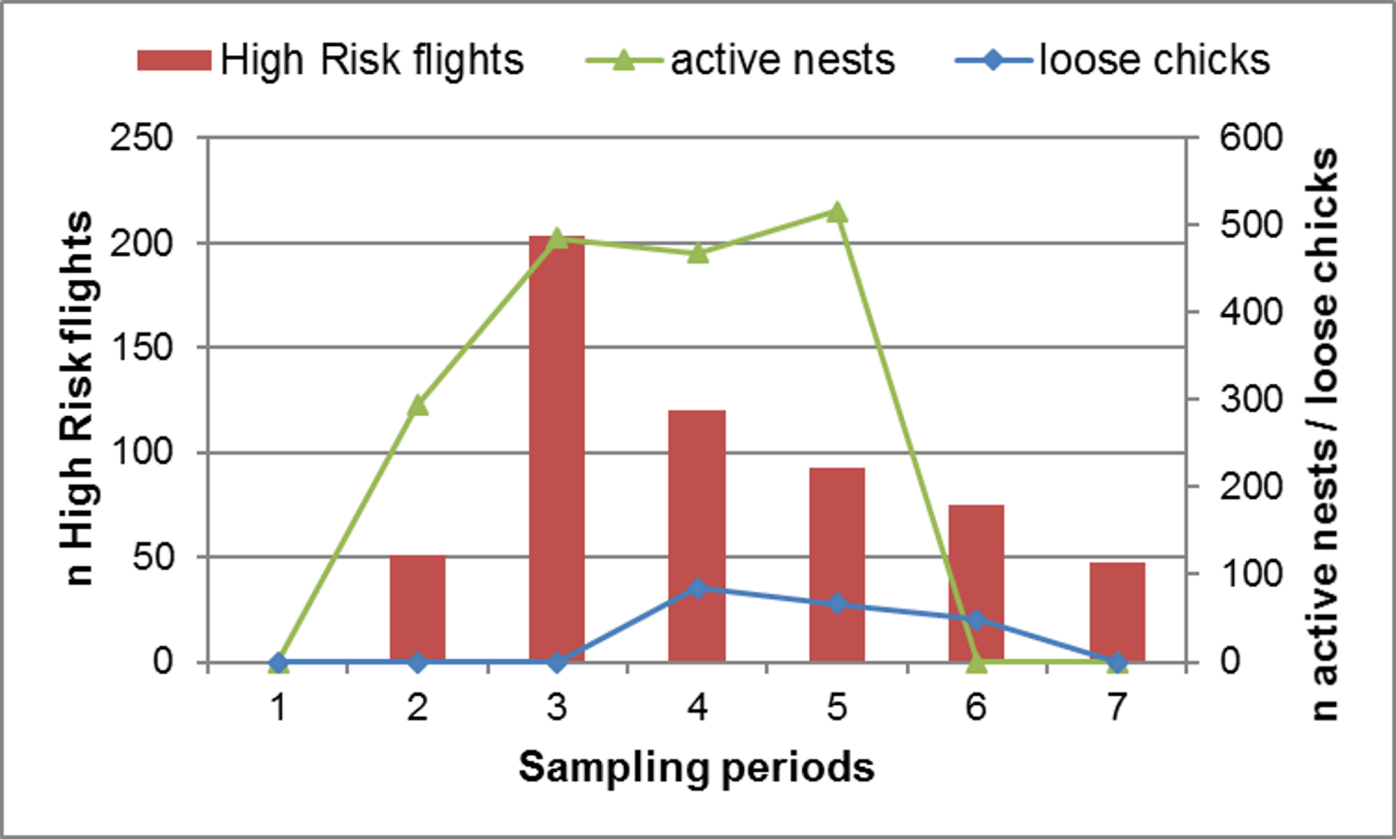
High risk flights by breeding pelicans. The frequency of High Risk flights through the wind farm development area in relation to the pelican breeding cycle on Dassen Island in 2014 (adapted from M. van Onselen unpubl. data).

Observer- and radar-based data show clearly that the incursion of pelicans into the proposed turbine layout was considerable, and regularly involved a significant proportion of the Dassen Island population. All indications are that volumes of pelican traffic vary seasonally, and correspond closely with the numbers of pelicans present on the island, with the intensity of breeding activity prompting provisioning birds to commute regularly between the island and feeding areas located mainly to the south (Fig 11).

Similarly, all the information collected shows that provisioning pelicans fly predominantly on a south-east/north-west axis (Figs 2 and 3), to and from feeding areas located around the urban centre of Cape Town. The main feeding site is the Vissershok Waste Management Facility, with as much as 25% of the local population present at this site at any one time during the 2013 breeding season. There are many other feeding sites to the south of Dassen Island that presumably play a secondary role in drawing the birds in this direction [17, 18], so closure or relocation of the waste management facility is probably not a practical or effective way to shift the pelicans’ preferred fly-way away from the wind farm.

Other indications of pattern in the movements of pelicans through the wind farm site may present more practical alternatives for impact mitigation. In addition to the clearly seasonal nature of this movement, the radar data in particular suggest strong temporal and conditional patterns too. Radar coverage showed that there were no pelican flights through the area at night. During the day, collision risk peaked in the early afternoon, probably reflecting the time it takes for pelicans departing either the island or feeding areas to reach the wind farm site, but also possibly related to the birds’ dependence on thermals for efficient cross-country flight [33], given that thermals are more prevalent during the warmest part of the day.

Perhaps most importantly, the pelicans moved through the area differently under varying wind conditions, with collision risk greatest in strong north-westerly and south-easterly winds (Figs 3 and 5), and lowest in lighter easterly or westerly breezes. This unfortunate tendency to by-pass the development area in calmer, warmer conditions (when the wind farm would be generating relatively little power), and to approach it in windier (and often cooler) conditions (when the wind farm would be generating at or close to capacity), might be compounded if the ground speeds of commuting birds are reduced by strong headwinds, exposing the birds to a heightened risk of colliding with turbine blades rotating at maximum speeds.

### Collision rates and population-level impacts

Estimates presented here of the frequency of High Risk flights by pelicans in relation to the proposed turbine layout are based on sufficiently large samples of spatially accurate data to be considered reliable. Similarly, variation in the severity of collision risk presents quite simple spatial and temporal patterns, and more subtle patterns in relation to environmental conditions, that are linked to the biology of the pelican population.

However, the conversion of collision risk data to mortality rates requires knowledge of the pelicans’ reaction to the placement of the wind turbines along their preferred flight path [3, 5]. Obviously, this knowledge is not yet available, and completion of these calculations demands that assumptions be made and various possible scenarios investigated. This approach was adopted here (Table 3), and despite relative certainty around exposure to the risk of collision, variation in postulated annual fatality rates remains considerable, with possible impact ranging from from negligible to extreme. While the actual mortality rate is likely to approximate the more moderate of these estimates, there is no guarantee that this will be so [34]. For example, should the avoidance rate be even slightly lower than 95% [28], mortality rates could exceed even the upper limits of the values postulated here (Tables 2–4). Hence, the outcomes of the collision rate modelling were inherently compromised and limited, in spite of the thoroughness and accuracy of the contributing flight path data.

Translating mortality rates into demographic impacts is also constrained by the reliability of the population parameters used. In this instance, there are some important great white pelican life history variables that remain poorly known (S2 Table). This deficiency, coupled with the susceptibility of the the Dassen Island pelican population to marked fluctuations in both size and breeding performance, brings uncertainty to the predictions of simple demographic models. Hence, confidence around the generated estimates of population-level impacts is low, and the value of these modelled trends remains questionable. However, while acknowledging these shortcomings, there were some useful demographic data available for what is a small, discrete and relatively well-studied population [17, 18], lending some credibility to the results presented. In fact, this study may be unique in combining representative quantities of accurate flight path information with relatively good, long-term demographic data in a pre-construction assessment of the possible impacts on birds of a proposed wind energy development.

The instability of the Dassen Island pelican population seems to be largely the product of its response to changes in the availability of artificial food sources [18]. This strong influence of anthropogenic factors, coupled with the knock-on effects of inflated pelican numbers on the conservation status of other threatened species [35], could suggest an opportunity to downplay the pelican mortality rates described here as simply a means of restoring natural balance in the local system. However, the affected pelican population is critical to the regional conservation of a species only recently up-listed to nationally ‘Vulnerable’ [36]. In this context, the Dassen Island great white pelicans should be considered as a conservation priority, and any potentially unsustainable impacts on this population should be viewed in a very serious light.

#### Impact mitigation scenarios

Despite these concerns, there are mitigation options available that, at least theoretically, could still render this wind farm project sustainable. The one most likely to be effective is simply to not install the highest risk turbines. This approach is consistent with the findings of many post-construction studies of collision impacts at wind farms, in which the vast majority of mortalities occur at only a few turbines [37, 9]. An effective remedy in most such cases is the retrospective shut-down of problem turbines [9]. In this case, the five most problematic turbines are located on slopes and/or high ground at points where pelican flocks habitually fly closest to the ground, and are particularly concentrated along the southern and south-eastern fringes of the proposed layout (Fig 3).

The modelled outcomes of a more “operational management” approach to impact mitigation, involving seasonal and/or temporal and/or conditional shut-downs of problem turbines, are less persuasive. The most pragmatic option appears to be to shut down the five highest risk turbines for about 6.3% of the year (six hours per day over three months–Table 4). While this approach has the considerable advantage of retaining all the turbines and hence the full generating potential of the plant, the predicted curtailment time requirements are already quite substantial, and given that the confidence around these estimations is relatively low, these shut-down times may not be sufficient in the final analysis.

Hence, even with the benefit of large quantities of spatially accurate data describing pelican flight patterns through the proposed wind farm, and relatively accurate demographic information, our estimates of possible collision mortality rates, and the affects these might have on the local pelican population, are necessarily limited by the assumptions we had to make about pelican responses to wind turbines [5, 6], and remaining uncertainties around the dynamics of the study population. In reality, the effect of the wind farm on the Dassen Island pelican population could be negligible, or it could result in rapid, regional extinction, and our results are not sufficiently reliable to confidently underwrite sustainable development.

## Supporting information

S1 Fig Variation in the size of the breeding population of great white pelican at Dassen Island over the last 20 years [38; M. van Onselen pers. comm.]. (PDF)

S2 Fig Timing of great white pelican movements to and from the Dassen Island colony on 07 November 2013. (PDF)

S1 Table Input parameters for the Band collision risk model [3] for great white pelicans at the proposed wind farm site on the Cape west coast. (PDF)

S2 Table Life history parameters of female great white pelicans used in the Dassen Island matrix population models. The number of female chicks reared is derived from the overall productivity of 0.12–0.42 chicks raised.pair^-1^. year^-1^ (de Ponte Machado 2007), multiplied by the sex ratio and then the proportion of the population that was breeding.
(PDF)

S3 Table Observation times and numbers of great white pelicans recorded flying through or close to the proposed development area over six sampling periods from July 2013-March 2014. Mean flock sizes given correspond to the flock size categories identified in the radar data.
(PDF)

S4 Table Radar survey time and tracking samples accumulated at the proposed Cape west coast wind farm study site over the period of July 2013 –March 2014. (PDF)

S5 Table GLM outputs for the volume of pelican traffic within the study area. Fixed effects are the terms expected to influence the numbers of pelicans observed. Random terms were added for Date (sd = 2.30) and SampleID (sd = 2.04). Estimates are changes on the log scale from the base Intercept term (Sampling period 2, fTime ≤ 09h00, fWindDirection = NE, mean WindSpeed = 3.12 m.s^-1^ and mean Temp = 18.75°C). Estimated Response is converted back to the response scale (pelicans.10 min^-1^) by taking the exponential of the Estimate, and is the estimated change in the mean for each fixed factor while others are being held constant*. For example, the estimated response shown for Sampling period is pelicans.10 min-1 when fTime is < 09h00, the wind is from the NE, and wind speed and temperature are at their mean values.
(PDF)

S6 Table The probability of High Risk flights occurring at each turbine in relation to the six sampling periods. “X” denotes the sampling periods during which the probability of High Risk flights was > 0.01, while “x” indicates other times when High Risk flights were made.
(PDF)
