# Supplementary figures and images for: Combining radar and direct observation to estimate pelican collision risk at a proposed wind farm on the Cape west coast, South Africa
Source: PLoS One. 2018 Feb 6;13(2):e0192515. doi: 10.1371/journal.pone.0192515 (PMC5800659; doi:10.1371/journal.pone.0192515)

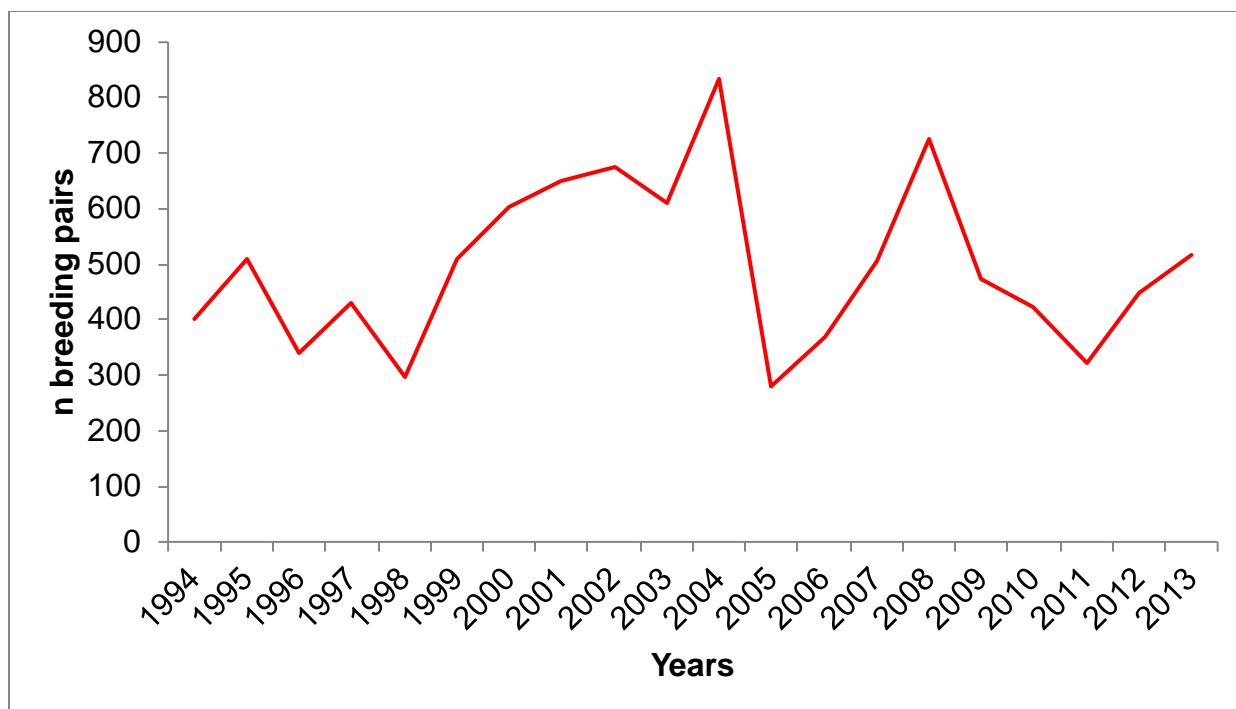

Supplement: S1 Fig — (PDF) [file pone.0192515.s001.pdf]

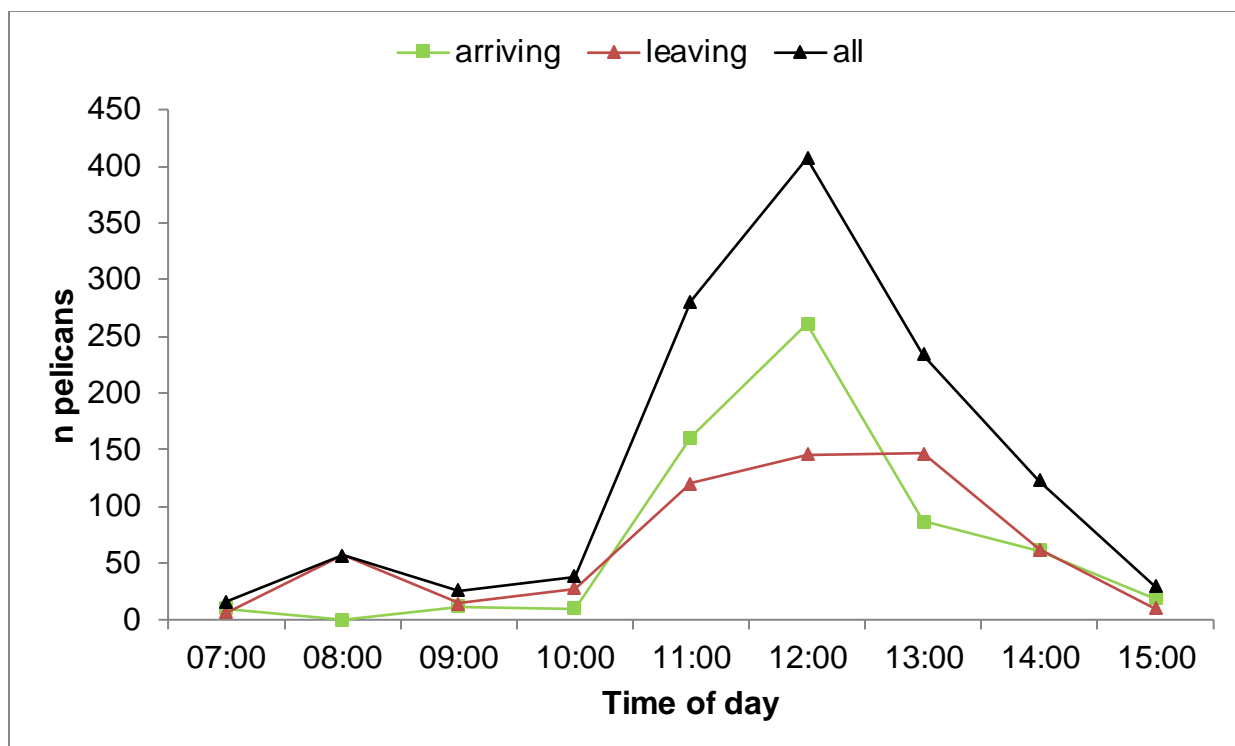

Supplement: S2 Fig — (PDF) [file pone.0192515.s002.pdf]
