# Supplementary material for: Combining radar and direct observation to estimate pelican collision risk at a proposed wind farm on the Cape west coast, South Africa
Source: PLoS One. 2018 Feb 6;13(2):e0192515. doi: 10.1371/journal.pone.0192515 (PMC5800659; doi:10.1371/journal.pone.0192515)
Supplement: S1 Table — (PDF) [file pone.0192515.s003.pdf]

**Parameter**	**Value(s) used**	**Data and source**
**BIOLOGICAL**		
Bird length	162 cm	Female 148cm, male 175 cm [47]
Wingspan	293 cm	Female 226 cm, male 360 cm [47]
Bird speed	3.2, 12.3, 22.0 $m.s^{-1}$	Average 44.1 $km.h^{-1}$ (range 11.6-79.2 $km.h^{-1}$, *n* = 589 High Risk flights from this study)
**TECHNICAL**		
Number of blades	3	
Rotor diameter	126 m	
Rotation period	3.6, 5.5, 11.3 s	Range = 5.3-16.5 rpm
Maximum chord width	4 m	
Average pitch angle	15°	Range = -9.5-90°
