# Supplementary material for: Combining radar and direct observation to estimate pelican collision risk at a proposed wind farm on the Cape west coast, South Africa
Source: PLoS One. 2018 Feb 6;13(2):e0192515. doi: 10.1371/journal.pone.0192515 (PMC5800659; doi:10.1371/journal.pone.0192515)
Supplement: S2 Table — The number of female chicks reared is derived from the overall productivity of 0.12–0.42 chicks raised.pair-1. year-1 (de Ponte Machado 2007), multiplied by the sex ratio and then the proportion of the population that was breeding. (PDF) [file pone.0192515.s004.pdf]

Parameter	Value(s) used	Data and source
**Female population size**	Approximately 1150 females	Monogamous so assume equal sex ratio [48]; up to 516 nests on Dassen 2013, therefore 1032 breeding adults (Van Onselen, Unpubl. Data); *ca* 45% of adults breed annually [22], therefore total = about 2300 adults or 1150 females
**Maximum age**	30	28 and 36 yr in Walvis Bay, 39 yrs in Eastern Cape [22]; at least 26, perhaps >28 yr [48]
**Maximum breeding age**	30	No data
**% population breeding**	34%, 46%, 57%	34-57% [22]; highly variable [48]
**Broods**	1-3 eggs, usually 2. Can lay replacement clutch after loss	[22, 47, 48]
**Age of first breeding**	3	[22]
**Sex ratio of eggs laid**	50:50	No data, assume equal
**Female chicks reared per female in the population**	0.02, 0.06, 0.12 (chicks x proportion breeding)	Obligate siblicide, so between 0.12-0.42 chicks raised.pair^{-1}. year^{-1} [22]
**% first year mortality**	26%	[22]
**% adult mortality**	4%	[22]
