# Supplementary material for: Combining radar and direct observation to estimate pelican collision risk at a proposed wind farm on the Cape west coast, South Africa
Source: PLoS One. 2018 Feb 6;13(2):e0192515. doi: 10.1371/journal.pone.0192515 (PMC5800659; doi:10.1371/journal.pone.0192515)
Supplement: S3 Table — Mean flock sizes given correspond to the flock size categories identified in the radar data. (PDF) [file pone.0192515.s005.pdf]

**Sampling period**	**Vantage point**			**Co-observation**			**Mean flock size (*n*)**	
	observation time (hh:mm)	*n* flocks	*n* birds	observation time (hh:mm)	*n* flocks	*n* birds	2-10 birds	>10 birds
1	17:37	23	323	-	0	0	6.0 (14)	41.0 (11)
2	20:38	75	856	22:44	79	1454	5.3 (79)	25.2 (75)
3	19:01	54	428	12:41	56	730	4.5 (72)	21.9 (38)
4	29:01	52	271	11:13	33	274	4.2 (69)	15.6 (17)
5	15:14	14	79	07:03	14	103	3.7 (23)	19.6 (5)
6	13:56	0	0	11:31	7	21	3.0 (7)	-
TOTAL	115:27	218	1957	65:12	189	2582	4.6 (263)	23.1 (144)
