# Supplementary material for: Combining radar and direct observation to estimate pelican collision risk at a proposed wind farm on the Cape west coast, South Africa
Source: PLoS One. 2018 Feb 6;13(2):e0192515. doi: 10.1371/journal.pone.0192515 (PMC5800659; doi:10.1371/journal.pone.0192515)
Supplement: S4 Table — (PDF) [file pone.0192515.s006.pdf]

**Sampling period**	**Survey time (hh:mm)**				**Tracked airborne wildlife**	
	Daylight	Overnight	Total	*n* 10 min observation periods	*n* track points	*n* tracks
1	51:59	23:40	75:39	584	319735	5247
2	73:43	34:27	108:10	679	416664	19852
3	65:54	29:39	95:33	788	350921	20186
4	70:12	26:36	96:48	637	157987	6417
5	67:28	28:06	95:34	773	331472	19293
6	65:37	31:06	96:43	601	179783	13181
Dassen	20:13	-	20:13	-	31187	1512
TOTAL	415:06	173:34	588:40	4062	1787749	85688
