# Supplementary material for: Combining radar and direct observation to estimate pelican collision risk at a proposed wind farm on the Cape west coast, South Africa
Source: PLoS One. 2018 Feb 6;13(2):e0192515. doi: 10.1371/journal.pone.0192515 (PMC5800659; doi:10.1371/journal.pone.0192515)
Supplement: S5 Table — Fixed effects are the terms expected to influence the numbers of pelicans observed. Random terms were added for Date (sd = 2.30) and SampleID (sd = 2.04). Estimates are changes on the log scale from the base Intercept term (Sampling period 2, fTime ≤ 09h00, fWindDirection = NE, mean WindSpeed = 3.12 m.s-1 and mean Temp = 18.75°C). Estimated Response is converted back to the response scale (pelicans.10 min-1) by taking the exponential of the Estimate, and is the estimated change in the mean for each fixed factor while others are being held constant*. For example, the estimated response shown for Sampling period is pelicans.10 min-1 when fTime is < 09h00, the wind is from the NE, and wind speed and temperature are at their mean values. (PDF) [file pone.0192515.s007.pdf]

**Model**	**Estimate**	**Std. Error**	***z* value**	**Pr (>|z|)**	**Estimated response**		
Fixed effects:					response	ci lower	ci upper
(Intercept)	1.91	0.87	2.20	0.03 *	6.73	1.23	36.87
Sampling period3	-3.35	1.26	-2.65	0.01 **	0.24	0.00	15.40
Sampling period4	-4.57	1.27	-3.60	<0.01 ***	0.07	0.00	4.60
Sampling period5	-7.67	1.30	-5.89	<0.01 ***	0.00	0.00	0.22
Sampling period6	-6.77	1.31	-5.18	<0.01 ***	0.01	0.00	0.55
Sampling period1	-3.23	1.26	-2.57	0.01 *	0.27	0.00	17.19
fTime>15h00	-1.51	0.20	-7.45	<0.01 ***	1.48	0.18	12.09
fTime09h00-12h00	1.24	0.19	6.38	<0.01 ***	23.31	2.90	187.15
fTime12h00-15h00	0.63	0.23	2.76	0.01 **	12.59	1.47	107.65
fWindDirectionNW	0.81	0.19	4.15	<0.01 ***	15.06	1.88	120.72
fWindDirectionSE	0.16	0.22	0.73	0.47 n.s.	7.90	0.94	66.71
fWindDirectionSW	0.29	0.22	1.35	0.18 n.s.	9.00	1.07	75.40
I(WindSpeed - mean(WindSpeed))	0.30	0.04	8.24	<0.01 ***	9.07	1.54	53.38
I(Temp - mean(Temp))	0.39	0.03	14.92	<0.01 ***	9.89	1.72	57.04
