# Supplementary material for: Combining radar and direct observation to estimate pelican collision risk at a proposed wind farm on the Cape west coast, South Africa
Source: PLoS One. 2018 Feb 6;13(2):e0192515. doi: 10.1371/journal.pone.0192515 (PMC5800659; doi:10.1371/journal.pone.0192515)
Supplement: S6 Table — “X” denotes the sampling periods during which the probability of High Risk flights was > 0.01, while “x” indicates other times when High Risk flights were made. (PDF) [file pone.0192515.s008.pdf]

**Turbine**	**Sampling period**					
	1	2	3	4	5	6
14		X				
15			X	x	X	X
16						X
18		X	X		x	x
20		x			x	x
23		X				
24		x			x	X
27		X	x	X		
28		x		X		x
30	x	x	x		x	
31	X	X	x	x	X	
32	x	x				x
33	x	X	X	x	x	
34	X	x		X	x	x
35	x	X	x	x	x	
